# Drivers of current and future anaerobic digester investment on US dairy farms

**DOI:** 10.3168/jdsc.2026-1066

**Published:** 2026-07-06

**Authors:** Jasmin Wehner, Wendong Zhang, Christopher A. Wolf, Lauren Ray, David McLaughlin

**Affiliations:** 1 Dyson School of Applied Economics and Management, Cornell University, Ithaca, NY 14853; 2 PRO-DAIRY, Cornell University, Ithaca, NY 14853; 3 Environmental Defense Fund, New York, NY 10010

## Abstract

Existing research on anaerobic digesters leaves operator motivations and future investment intentions largely unexplored. We address this gap by providing empirical evidence from a survey of 64 dairy digester operators with 164,639 milk cows across 11 US states, conducted in 2025. Half of all digesters in our sample were managed by third-party developers or firms, and nearly half of operators reported an intent to build new or reinvest in existing systems. Although cost share programs and financial incentives were identified as the most important factors for both current and prospective installations, labor availability and regulatory certainty were identified as additional barriers to future digester adoption. Our findings suggest that cost share programs and targeted support mechanisms for digester upgrades are key for successful digester adoption. Contrary to prior literature, we find that expansion plans were driven by expected profits and next-generation interests rather than digester net revenues. Notably, 95% of digester operators did not increase herd sizes because of the digester.

## Graphical Abstract

**Figure:**
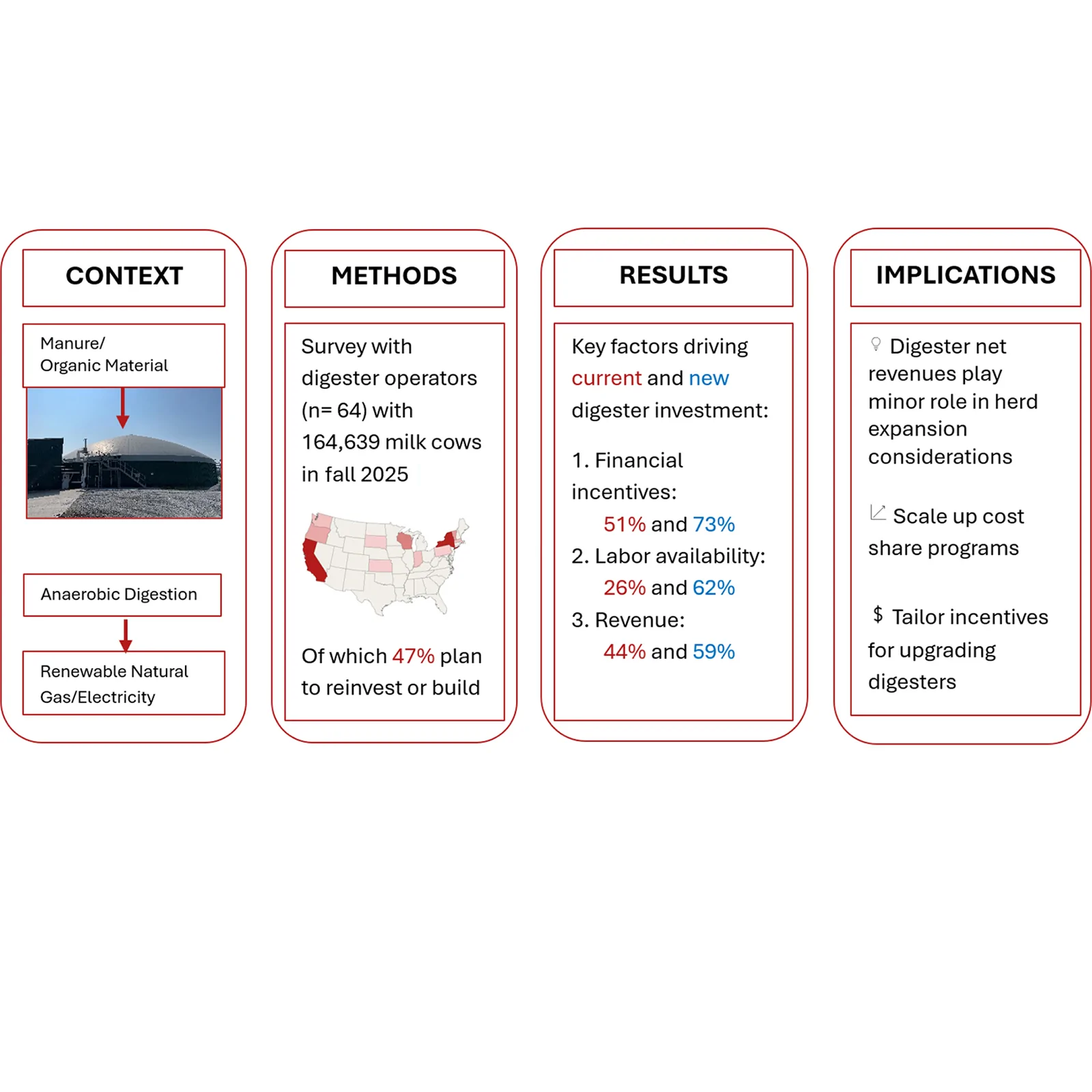

Livestock agriculture is a substantial source of greenhouse gas emissions, with manure management and enteric fermentation representing the 2 largest contributors (US EPA, 2022). Among available manure management practices, anaerobic digestion offers the highest relative methane reduction potential, making it a central emission reduction tool in the US dairy sector (US EPA, 2024a, 2025). Anaerobic digesters (**AD**) achieve this by converting volatile solids in manure into biogas, which can subsequently be upgraded to pipeline-quality methane, also known as renewable natural gas (**RNG**), or used directly for electricity generation (Rotz et al., 2025). Federal and state credit and cost share initiatives have played a central role in shaping overall investment decisions in dairy digester projects. Over the past decade, financial incentives such as the federal Renewable Fuel Standard and state-level low-carbon fuel standard (**LCFS**) programs have made RNG production designated for transportation fuel a more lucrative pathway than electricity generation (California State Legislature, 2016; Lazenby, 2022; O'Hara et al., 2023; CDFA, 2025). At the same time, the rising share of third-party management and ownership, massive capital costs, and labor constraints have influenced how digesters are financed and managed. Accordingly, the objective of this paper is to provide empirical evidence on current and future motivations and barriers related to dairy manure digester installations.

We hypothesize that, first, financial incentive programs, especially cost share funding and credit-based revenue opportunities (e.g., California's LCFS, or renewable identification numbers [**RIN**]), are the primary determinants of both current and future digester adoption among US dairy farms. This study examines the importance of related factors, such as labor availability and surrounding energy infrastructure, on adoption decisions. We hypothesize that, compared with past digester adoption decisions, future digester investment decisions are increasingly constrained by concerns about labor availability and regulatory uncertainty. We also hypothesize that structural differences exist between California and non-California operations, including digester age and ownership structure (i.e., whether the digester is operated by the farm or by a third-party company). To examine these hypotheses, we report findings from a nationwide survey of dairy farmers with AD experience, focusing on operators' perceptions of the policy instruments designed to incentivize digester installation.

These results complement existing literature by documenting motivations and barriers associated with both current and prospective digester installation decisions across the United States. We expand the most recent AgStar digester database from the US Environmental Protection Agency by capturing detailed information on motivations behind current and potential future digester installation, investment structures, and revenue streams (US EPA, 2024a). We build from state-level case studies that emphasized the role of policy frameworks in enabling successful digester adoption by providing nationwide evidence (Wang et al., 2011; Pierce and Strong, 2023). In addition, our analysis contributes to ongoing discussions regarding the relationship between herd size expansion and digester installations (California Air Resources Board, 2024; Magesh et al., 2025) as well as the extent to which surrounding energy infrastructure shapes installation decisions (O'Hara and Xiarchos, 2023). The survey results may be useful in designing effective and targeted financial incentive mechanisms to accelerate digester installations across the United States.

The survey was administered by the Cornell Center for Conservation Social Sciences from September to December in 2025, tested with dairy farmers, and scrutinized by academic experts. We identified dairy farmers with digester experience using records from the US Environmental Protection Agency and complemented the list with state-specific sources (US EPA, 2024a; J. Wehner, C. Wolf, and W. Zhang, unpublished data). Inclusion criteria were that the dairy farm AD was operational at the time of survey completion or that the operator had previous experience with an AD. The project was approved by Cornell University's Institutional Review Board (approval #IRB0149938). The initial survey and cover letter were sent with a symbolic $2 bill to encourage participation, followed by a reminder letter 1 wk later. A second survey was mailed 3 wk after the initial contact, with a final reminder sent another week later, following survey method recommendations in Dillman et al. (2014) and Slijper et al. (2026).

After accounting for 67 undeliverable addresses, 352 surveys were delivered, resulting in 64 usable responses, an 18% response rate. The majority of responses, 80%, were submitted by mail, and 20% were submitted online. Our responses account for 15.8% of all digesters in operation or construction (US EPA, 2024a). The 64 responding farms collectively reported 164,639 milk cows, providing substantial industry coverage given that farms with digesters operate at far above-average scale. Some questions were asked with a Likert scale response such as 1 = very unimportant to 5 = very important. We summarized survey responses by calculating summary statistics excluding the share of people who did not answer specific questions. Depending on the variable structure, we compared means using appropriate statistical tests with significance determined at *P* < 0.05 and calculated correlations with the Pearson's r test using the programming language R version 4.5.2 (R Core Team, 2025).

Summary statistics of respondents are displayed in Table 1. State-level responses reflect important dairy-producing states and installed digesters, led by California (30%), New York (29%), and Wisconsin (11%; O'Hara and Xiarchos, 2023; US EPA, 2024a). Dairy farmers reported having on average 2,613 (SD 1,903) milk cows on Jan. 1, 2025, which is consistent with dairy farm sizes with digester experience (O'Hara and Xiarchos, 2023) but much larger than the averages across all US dairy farms (380 milk cows; USDA-NASS, 2022). Annual milk production per cow was 12,777 kg, which exceeds the average US milk production per cow by 14.67% but is consistent with productivity of larger dairy herds (USDA-NASS, 2026). Average primary dairy farm operator age was 52 yr, almost identical to the general average primary dairy farm operator age (USDA-NASS, 2022).

**Table 1:** Dairy farm anaerobic digester survey summary statistics; where applicable, statistical test results comparing the survey sample to the AgStar database are reported below each category

Category	Variable	Digester survey (SD)	EPA AgStar
Operator characteristics			
Region	Midwest (IN, SD, KS, WI)	17%	17%
	Northeast (NY, MA, PA, VT)	42%	22%
	West (CA, OR, WA)	41%	39%
χ^2^(2) = 4.76, *P* = 0.09			
Farm	Milk cows (head)	2,613 (1,908)	4,322 (5,361)
t(232.3) = 4.92, *P* <0.001			
	Milk per cow (kg/yr)	12,776 (1,818)	—
Age	Operator (yr)	52 (12)	—
	Dairy farm (yr)	55 (36)	—
Education	High school diploma or less	27%	—
	Technical training	20%	—
	College degree	44%	—
	Graduate degree	9%	—
Income from dairy	Less than 25%	3%	—
	26–50%	3%	—
	51–75%	6%	—
	Over 75%	88%	—
Digester characteristics (values based on year 2024)			
Digester presence	One digester	80%	—
	More than 1 digester	9%	—
Digester age		10 (7) yr	10 (8) yr
t(82.05) = 0.71, *P* = 0.48			
Installation	Before 2017	52%	52%
t(71.92) = 0.58, *P* = 0.57			
Management	Developer	50%^1^	95%^2^
t(66.08) = 7.09, *P* <0.001			
Digester type	Complete mix	36%	25%
	Covered lagoon	34%	37%
	Plug-flow	17%	32%
	Other	5%	5%
χ^2^(3) = 6.67, *P* = 0.08			
Biogas end use	RNG off-farm	45%	47%^3^
	On-farm	3%	
	Electricity off-farm	30%	21%^3^
	On-farm	14%	
	Collected and flared	9%	3%
χ^2^(3) = 9.12, *P* = 0.03			
Co-digestion	Share	19%	24%
t(83.99) = 0.92, *P* = 0.35			
Pipeline distance	(in miles)	9.97 (18.48)	—
Biogas production	(in SFCM^4^)	61,139 (99,505)	—
N		64	478

1 Developer defined as manager or operator of the digester.

2 Developer defined as system designer or developer and affiliates.

3 No distinction made between on- or off-farm use.

4 SFCM = standard cubic feet per minute.

We display digester characteristics in Table 1 and, where applicable, compare them to the digester characteristics of the AgStar database (US EPA, 2024a). Comparison of our survey results against AgStar provides a reasonable indication of the representativeness of our results, given that this database represents the most widely used and comprehensive resource available for this purpose.

The dairies from the survey have 2,613 milk cows on average, which is a smaller herd size than reported in AgStar (Welch's t-test [232.3] = 4.92, *P* < 0.01). The regional composition of our survey sample closely mirrors AgStar data, with the Western region being the most represented, followed by the Northeast and the Midwest (χ^2^[2] = 4.76, *P* = 0.09). Covered lagoons were the most common digester type in the AgStar database (37%) and the second most common type in the survey (34%). Complete mix digesters were more prevalent in our survey than in AgStar data (36% vs. 25%). Plug-flow digesters, by contrast, were more represented in AgStar than in our survey (32% vs. 17%). Despite these distributional differences, the samples were not statistically distinguishable in terms of digester type (χ^2^[3] = 6.67, *P* = 0.08)

On average, digesters had been in operation on the dairy farms for 10 yr in both the survey and AgStar data (Welch's *t*-test [82.05] = 0.71, *P* = 0.48). The survey responses mirror broader trends in digester development on US dairy farms: digesters located in California were built more recently than digesters outside California (5.6 vs. 10.6 years, Welch's t [47.55] = 3.40, *P* < 0.01) and were significantly more likely to be developer-managed than those outside California (χ^2^[1] = 12.29, *P* < 0.001).

Policy incentives have played a central role in shaping AD adoption, most notably California's Senate Bill 1383 (SB 1383), passed in 2016 and framed as a set of emission reduction targets. Effectively, it created a quasi-mandate for emission reductions on dairy farms operating in the state, incentivizing a structural shift in biogas end use from electricity generation to RNG production, facilitated through California's LCFS. Because dairy farms located outside California were also eligible to earn credits under California's LCFS, the program carried implications for AD adoption nationwide (California State Legislature, 2016; Lazenby, 2022). Accordingly, the survey responses show a shift toward predominant biogas end use. Before 2017, electricity sold off-farm was the most common biogas end use, accounting for 36% of digesters installed before that year, compared with only 24% producing RNG sold off-farm. Among digesters installed from 2017 onward, the share producing RNG sold off-farm tripled to 76%, whereas the share selling electricity off-farm fell to 8%. Most digesters produce at least some biogas for off-farm use (75%), with no significant difference between digesters located in California or elsewhere (χ^2^[1] = 0.097, *P* = 0.76). We observed a significant correlation between herd size and biogas production for off-farm sale (*F*_1,61_ = 9.22, *P* < 0.001), suggesting that larger dairy operations are more likely able to afford capital-intensive RNG infrastructure (US EPA, 2024b).

The data further revealed a shift in digester ownership and management structure over time. Farmer-owned and managed systems dominated installations before 2017, but the majority of digesters built after 2017 were developer-owned and managed, reflecting the growing role of third-party developers in the sector. This pattern also varied by digester type: the majority of covered lagoons were developer-built and managed (86%), whereas roughly two-thirds of complete mix (61%) and plug-flow (63%) systems were farmer-built and managed. Developer-managed digesters were located significantly closer to natural gas pipelines than farmer-managed systems (Wilcoxon rank-sum test, W = 155.5, *P* < 0.01), suggesting that developers might strategically place installations in proximity to existing gas infrastructure. In our survey, half of respondents reported that their digester was managed by an outside firm. This compares to 95% of digesters in the AgStar database being associated with a system designer, developer, or affiliate. The substantial, significant (Welch's *t*-test [66.08] = 7.09, *P* < 0.01) difference between these numbers likely reflects a distinction in what each survey captured, as the AgStar database records broad third-party involvement across all stages of development and ownership, whereas our survey specifically asked about operational management.

Some digester systems process multiple organic materials, called co-digestion. The most common other materials reported for co-digestion are food waste and fats, oils, and grease in both our digester survey and the AgStar database (US EPA, 2024a). Co-digestion rates were comparable between our survey and AgStar data (Welch's *t*-test [83.99] = 0.92, *P* = 0.35). Concerning responses from the digester survey, the weight of these other materials averaged 5,786 (SD 7,952) tonnes per system. Accordingly, most digesters received tipping fees as compensation for accepting organic waste, at an average below $22.05 per tonne of organic waste. Total investment (including farm and any cost share or developer financing) was at median category between $1,000 to $1,999 per cow, and there was no significant difference in investment costs by ownership structure (Welch's *t*-test [41.99] = −0.65, *P* = 0.51). For the majority of digester operators, annual net digester revenues were mostly below $150/cow. Notably, developer-managed RNG digester operators reported the lowest annual net revenues per cow, predominantly below $99/cow, potentially reflecting revenue sharing arrangements with third-party operators. Most digesters generated variable revenues (52%), with fixed revenues reported by only 25% of respondents, most commonly for periods shorter than 10 yr.

Figure 1 compares the importance of various factors driving currently installed digesters versus potential new installations, a particularly relevant distinction given that 44% of respondents indicated plans to reinvest in or construct a new digester. Several financial incentives at federal and state level are available to support digester installation (Lazenby, 2022; CDFA, 2025), which were also prominent: 70% of respondents stated that cost share programs were important for their current digester, and 79% would not have built the digester without cost share. The majority of respondents (83%) viewed cost share programs as important or very important for new digester installations. The average cost share provided for digesters was 57% (SD 36%), and there was no significant difference in cost share between digesters built in California or outside California (Welch's *t*-test [29.89] = −0.07, *P* = 0.94), built before 2017 or after (Welch's *t*-test [34.28] = 0.6, *P* = 0.58), nor between farm- or developer-managed digesters (Welch's *t*-test [36.61] = −0.6, *P* = 0.5). Moreover, there was no significant correlation between cost share and herd size (Pearson's r = −0.17, *P* = 0.28).

**Figure 1:**
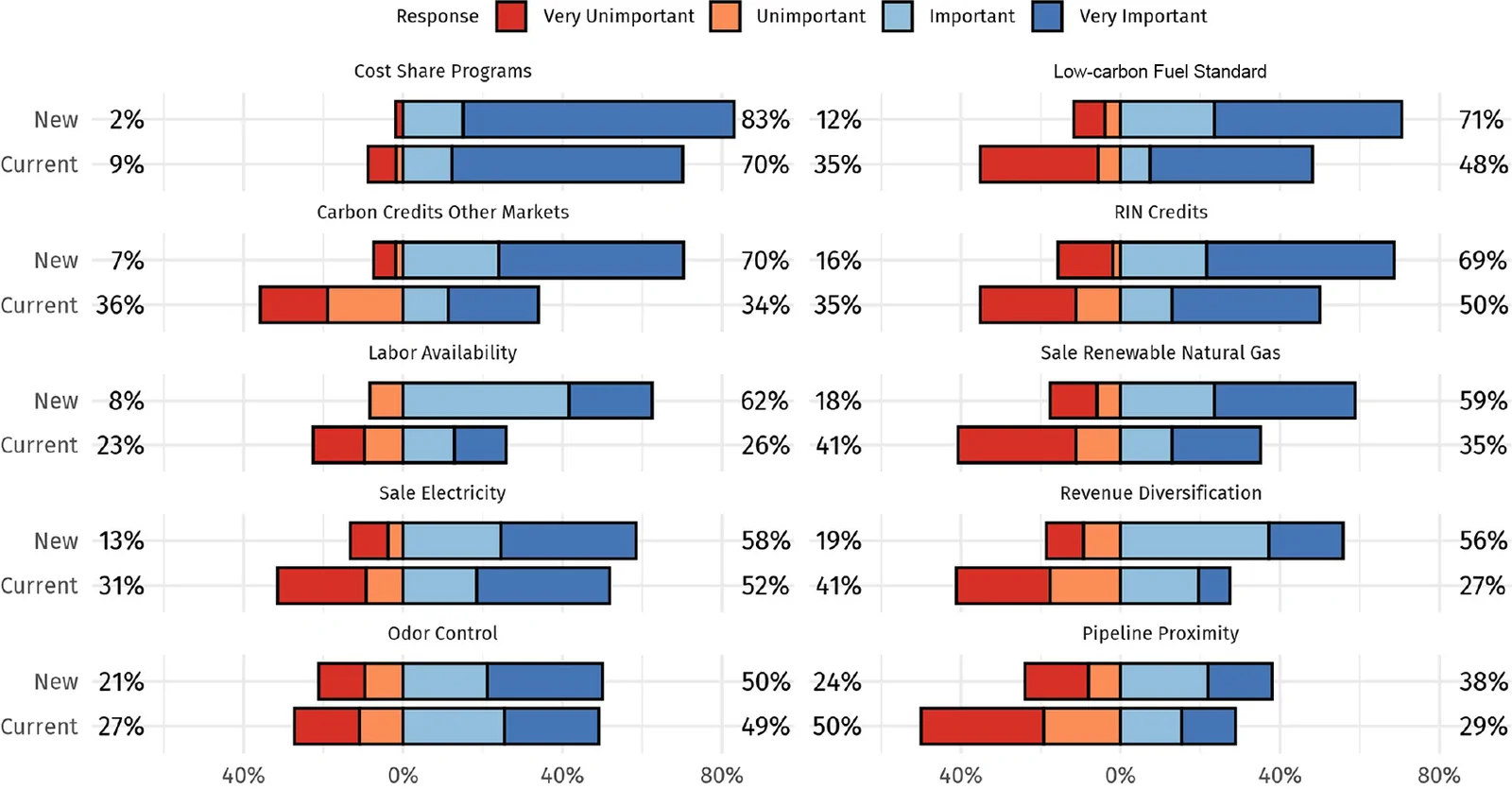
Share of respondents indicating whether a factor is unimportant (bars to the left) or important (bars to the right) for the decision to install current and potential new dairy digesters. The bars exclude the shares of the respondents who indicated to be indifferent respective factors. Visualization adapted from El Benni et al. (2025).

Other financial incentives, such as credits from California's LCFS, or RIN credits under the federal Renewable Fuel Standard program or elsewhere, become substantially more important for new digester installations than for current digester decisions. Revenue-related factors, such as the sale of RNG or electricity, were rated as important by respectively 35% and 52% for current digester installations. Interestingly, the importance of labor availability increased most between current and new digester installations, by a factor of 2.4, from 26% to 62%. This sharp increase may reflect the more specialized workforce required to operate modern digester systems compared with digesters of the older generation. The sale of RNG increased in importance while the importance of electricity sale remained relatively constant for new digesters. Other advantages associated with digesters, such as revenue diversification, were considered more important for prospective digester installations than for currently installed digesters. In contrast to qualitative evidence, odor control was only a minor motivation for digester installations (Pierce and Strong, 2023). Previous research has found that pipeline proximity, relevant for RNG-producing digesters, was associated with increased likelihood of digester installation in earlier periods of installations (J. Wehner, C. Wolf, and W. Zhang, unpublished data). However, pipeline proximity was viewed here as the least important aspect for current digester installations. Overall, the importance of pipeline proximity was valued as slightly higher by operators of RNG-producing digesters compared with non-RNG-producing digesters (Wilcoxon rank-sum test: W = 168.5, *P* = 0.0015) and by developer-managed digesters compared with farm-managed digesters (Wilcoxon rank-sum test: W = 229, *P* = 0.04). The importance of pipeline proximity did not change significantly for digesters installed before or after the year 2017 (Wilcoxon rank-sum test: W = 384, *P* = 0.18). Specific for new digester installations, regulatory uncertainty was a major consideration, with 61% of respondents indicating it was important or very important.

Nearly half (45%) of respondents indicated general plans to increase herd size by 2030, with most planning increases of up to 25% (n = 12) or between 26% and 50% (n = 5), and 2 respondents indicated expansion plans of more than 100%. Herd size expansion plans were driven by expected profits and next-generation interests (see Figure 2). Although some literature suggests that dairy digesters may be a driver for herd size expansion (Magesh et al., 2025), our survey responses indicated that digester net revenues (digester-related price and sales volumes) played, at best, a minor role (7.25%) in expansion decisions, ranking second to last among the factors farmers consider in their expansion decisions (see Figure 2). Three respondents indicated that their milking herd size would be 17% smaller on average without a digester, but the vast majority (95%) reported that they did not expand their herd because of the digester.

**Figure 2:**
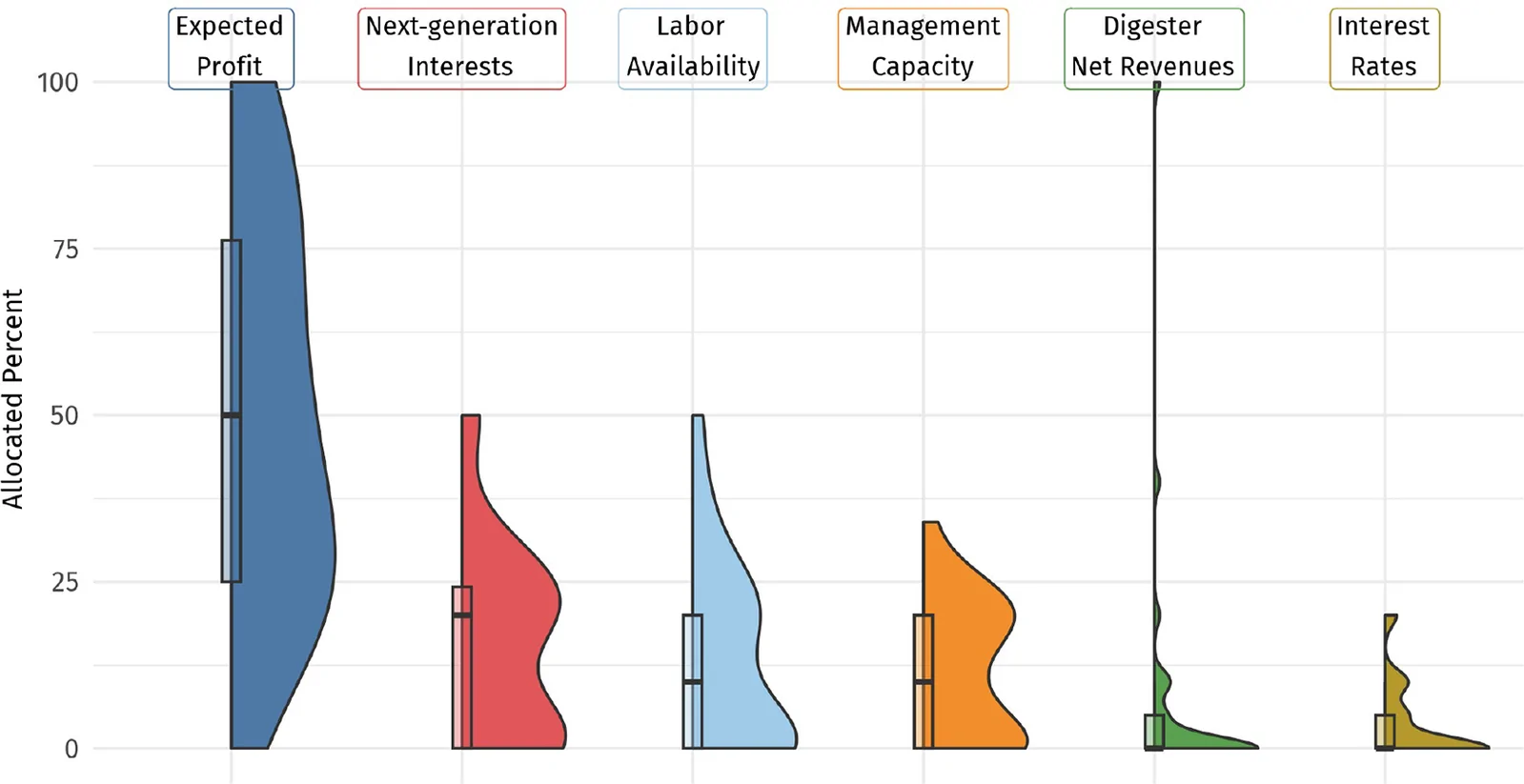
Relative importance of factors influencing herd expansion decisions. Respondents distributed 100% across 6 factors displayed as half-violin plots. Boxplots display the distribution of each variable. The horizontal line within each box represents the median, and the lower and upper edges of the box correspond to the 25th and 75th percentiles.

We acknowledge the possibility of nonresponse bias, given the absence of responses from states with both larger herd sizes (e.g., Idaho, Texas) and smaller herd sizes (e.g., Minnesota; USDA-NASS, 2026), as well as the growing role of developer companies that may reduce dairy operators' familiarity with digester-related topics. Additionally, the comparison to the AgStar database is subject to limitations, as AgStar data relies on voluntarily disclosed information and may not reflect current conditions. Similar trends of our nationwide survey with dairy digester operators and the AgStar database indicate that our results offer several actionable insights for policymakers, industry partners, and farm management teams seeking to expand the use and effectiveness of AD in the US dairy sector.

Additional digester investment is influenced by the availability of federal and state cost share programs, particularly those that support capital-intensive, first-generation digester investments. Regulatory uncertainty emerged as a major concern for future projects, with 61% of operators rating it as important or very important. This finding aligns with prior research suggesting that policy uncertainty around revenue streams and cost sharing arrangements acts as a barrier to digester investment (Lee and Sumner, 2018). The recent pause of one of the guaranteed loan programs (Rural Energy for America Program [REAP]), due to elevated loan delinquency rates, removes a federal guaranteed loan source at a time when regulatory uncertainty is already seen as barrier for digester investment decisions (Bienkowski, 2026; USDA Rural Development, 2026). Yet those delinquency rates may themselves be a result of that regulatory uncertainty, as less-predictable incentive streams cause project revenues to fall short and loan repayment to become increasingly difficult, as illustrated by fluctuations of California's LCFS credit prices and the repeated downward revision of RIN volume requirements (Gerveni et al., 2026; Smith, 2026).

The suspension of cost share support under these conditions risks amplifying an already challenging investment environment. Thus, greater long-term funding certainty, via multiyear appropriations or guaranteed program continuity, would reduce investment risk and encourage new entrants. Credit-based programs (LCFS, RIN), which the survey shows are increasingly important for future installations, could be refined to establish regulatory stability and clearer long-run pricing expectations.

Although earlier literature emphasized proximity to pipelines as an adoption driver in certain cases, this survey found that pipeline proximity was among the least important factors for current digester decisions and was only marginally more important for future projects (J. Wehner, C. Wolf, and W. Zhang, unpublished data). Localized biogas upgrading and virtual pipeline solutions appear to reduce infrastructure dependence for operators without direct pipeline access. Grants or loans for small-scale upgrading equipment, gas compression, or trucking systems could further democratize entry for farms distant from pipelines.

Nearly half of operators (44%) intend to reinvest in or construct a new digester, suggesting substantial momentum among the existing operator base. Incentive programs might consider creating dedicated tracks for reinvestment, recognizing the different needs of aging systems (e.g., equipment replacement, upgrading to RNG compatibility).

Finally, despite speculation in policy and academic circles, survey responses demonstrate that digester net revenues play a minimal role in herd expansion, and 95% of operators did not expand herds due to the digester. Digesters function as emission reduction tools across varying herd sizes, with adoption patterns indicating that incentives are not consistently associated with herd expansion.

In conclusion, this study documents recent developments in AD installations across US dairy farms based on a survey administered in autumn 2025 that reflects a balanced representation of installation years, digester types, and biogas end uses. Our findings are consistent with the hypothesis that financial incentives are the primary consideration for both current and prospective digester installations. At the same time, our results highlight labor availability and regulatory uncertainty as important constraints shaping future adoption decisions. Additionally, digester installation decisions appeared to be largely independent of herd size expansion plans. Given the substantial methane mitigation potential of AD adoption and the strong reinvestment intent observed in this survey, 2 factors are identified as relevant to investment outcomes: (1) long-term pricing structures, and (2) cost share programs that lower financial barriers for initial and ongoing investments.
